# Hypothesised cutaneous sites of origin of stage III melanomas with unknown primary: A multicentre study

**DOI:** 10.1002/ijc.34020

**Published:** 2022-04-25

**Authors:** Bethan Clayton, Ferhan Muneeb, Maria Celia B. Hughes, Megan E. Grant, Kiarash Khosrotehrani, B. Mark Smithers, Romina Spina, Luca G. Campana, Deemesh Oudit, Adele C. Green

**Affiliations:** ^1^ Department of Surgery The Christie NHS Foundation Trust Manchester UK; ^2^ Population Health Department QIMR Berghofer Medical Research Institute Brisbane Australia; ^3^ Molecular Oncology Group CRUK Manchester Institute, University of Manchester Manchester UK; ^4^ Experimental Dermatology Group The University of Queensland Diamantina Institute, Translational Research Institute Brisbane Australia; ^5^ Department of Dermatology Princess Alexandra Hospital Brisbane Australia; ^6^ Queensland Melanoma Project Princess Alexandra Hospital, The University of Queensland Brisbane Australia; ^7^ Department of Surgery Veneto Institute of Oncology IOV‐IRCCS Padua Italy; ^8^ Psychology Unit University Hospital of Padova Padova Italy; ^9^ Department of Surgical Oncological and Gastroenterological Sciences (DISCOG) University of Padova Padova Italy; ^10^ Faculty of Biology Medicine and Health, University of Manchester Manchester UK

**Keywords:** anatomic site, body sites, cutaneous melanoma, melanoma, unknown primary

## Abstract

Based on molecular evidence that melanomas with unknown primary (MUPs) arise from the skin, we hypothesised that sites of MUPs are disproportionately on trunk and lower limbs, sites that are not readily visible to patients and clinicians. We tested this hypothesis by inferring the anatomic site of origin of MUPs from the corresponding known cutaneous sites of melanoma patients with known primary tumours (MKPs). We analysed data from three separate cohorts of patients from Brisbane, Australia (n = 236); Manchester, UK (n = 51) and Padova, Italy (n = 33), respectively, who first presented with stage III melanoma with lymph node metastases. We matched two MKP patients to each MUP patient based on lymph node dissection (LND) site, age and sex, and imputed cutaneous sites of origin of MUPs from their two matched MKPs for study countries, giving two possible sites for each MUP per centre. Overall, results showed that MUP patients were predominantly male, and trunk was the most likely origin, comprising around a third to a half of MUPs across the three cohorts. The remaining MUP inferred sites varied by country. In the Australian cohort, the legs accounted for a third of imputed sites of MUPs, while in the UK and Italian cohorts, the most frequent site was the arms followed by the legs. Our findings suggest the need for regular and thorough skin examination on trunk and limbs, especially in males, to improve early detection of cutaneous melanoma and reduce the risk of metastatic disease at the time of presentation.

## Graphical abstract

What's new?

Molecular evidence points to the cutaneous origin of melanomas with unknown primary (MUPs). However, whether regressed or overlooked melanomas on specific cutaneous locations are associated with MUPs remains unclear. Assuming a skin origin, the authors imputed the likely sites of origin of stage III MUPs from the corresponding cutaneous sites of matched patients with known primary melanoma. The trunk was the most likely origin of MUPs, followed by the limbs, with males being more affected. The findings underscore the need for thorough skin examination of the trunk and limbs, especially in males, to reduce the risk of MUPs.

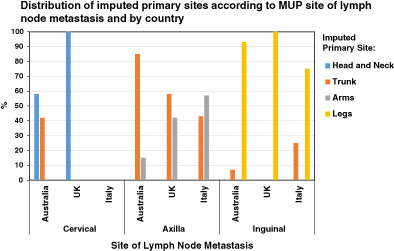

**Abbreviations**
LND: lymph node dissection
MKPs: melanoma patients with known primary tumours
MUPs: melanomas with unknown primary
UV: ultraviolet

## 1 INTRODUCTION

Most melanomas are cutaneous, arising from melanocytes or nevus cells in the skin. A small proportion of primary melanomas, around 5%, have extracutaneous origin such as the eye, and mucosal sites like the genitalia,^1^ while approximately 3% present as metastatic disease arising from an unknown primary site, termed ‘melanoma of unknown primary’ (MUP).^2^

MUP was first described in the early 20th century, comprising 2.4% of a hospital series of over 1000 melanoma patients treated in a large US hospital over three decades who presented with secondary melanoma without a discernible primary.^3^ Das Gupta et al^4^ subsequently specified criteria to be met before a diagnosis of MUP could be made, namely no evidence of: previous skin lesion excision with a scar in the region of the draining lymph node basin containing the metastatic melanoma; no past orbital surgery; and the conduct of a complete physical (skin, eye, anal and genital) examination. MUP presents mainly in regional lymph nodes, comprising up to 60% of MUPs in most (but not all^5^) studies, with the rest presenting as visceral deposits.^2^

Various hypotheses about the origins of MUP have been proposed. The main theory postulates that MUPs arise from skin but have regressed or been missed on clinical examination,^6^ and this is supported by mutation profiles studies showing very high somatic mutation rates with ultraviolet (UV) signature and high rates of BRAF and NRAS mutations consistent with melanomas arising on sun‐exposed skin.^1^ A less favoured explanation is that MUPs arise in ectopic melanocytes in internal organs^7^ but this theory would predict MUPs randomly distributed across age‐groups and by sex and is hard to reconcile with the findings of a systematic review^8^ showing that most MUPs occur in the 50 to 69 year age group and that males are twice as likely as females to be diagnosed with MUP. Furthermore, while most MUPs present as regional lymphadenopathy^2^ (clinical stage III) especially axillary, females notably present with inguinal node involvement more often than males, consistent with the known predilection of cutaneous melanoma for the legs of women.^9^

Thus strong empirical evidence, both epidemiological^2^ and genetic,^10^ points to the cutaneous origin of MUPs, but raises the question of whether melanomas on specific cutaneous locations are associated with MUPs. We postulated that MUPs most likely arise from sites like the back and lower limbs that cannot be seen directly or are not regularly inspected, so that suspicious pigmented lesions are overlooked. We investigated this by matching patients with stage III MUPs according to the site of initial lymph node metastasis, age and sex to patients with stage III melanoma arising from known cutaneous primaries (MKPs), and inferring the likely primary cutaneous sites of the MUPs from that of the known primary of the matched MKP patients.

## 2 MATERIALS AND METHODS

Three separate clinical records‐based studies of stage III nodal melanoma patients were conducted in centres in Australia, The United Kingdom and Italy, respectively.

### 2.1 Australia

Melanoma patients aged >18 years referred to the Princess Alexandra Hospital Melanoma Clinic, Brisbane, in 2000 to 2011 for regional lymph node dissection (LND) were identified for a study comparing survival in patients with stage III MUP and MKP as previously described^11^ and approved by institutional ethics committees (updated for the current study, P3610). We extracted demographic data and clinical details from medical records and collated these in a database. We included patients who had palpable regional lymph node metastases from melanoma, a negative staging total body CT scan, and underwent therapeutic cervical, axillary or inguinal LND.^11^ For MKP patients, the anatomical locations (head or neck, arm, leg and trunk) and thickness (in mm^2^) of the primary tumours were retrieved from histology reports. Patients were considered to have a MUP if a primary melanoma could not be clinically identified on the skin, ocular or other mucosal sites. Demographic characteristics (age at LND and sex) and site of LND were likewise extracted from histology reports. We used the macro for SAS software (SAS Institute Inc, Cary, North Carolina) developed by Mortensen et al^12^ to match two randomly selected MKPs (controls) to each MUP patient (case) according to sex, 5‐year age group and LND site (two controls per case being the maximum number feasible across all study databases).

### 2.2 The United Kingdom

The study was conducted at The Christie NHS Foundation Trust, a tertiary cancer treatment centre in Manchester, with approval of the institutional ethics committee (16/LO/0387). From a dedicated clinical database of melanoma patients treated between 2002 and 2016, we extracted details of all patients with stage III nodal MUP and examined their medical charts to confirm the diagnosis according to recommended diagnostic criteria.^4^ For every confirmed MUP patient, we aimed to select two MKP patients, matching for the same variables, namely sex, 5‐year age group and LND site.

### 2.3 Italy

The study was conducted at the Veneto Institute of Oncology, Padova, and approved by the institutional ethics committee (CESC IOV 2020/36). Patients treated for Stage III disease were identified from the prospective melanoma register activated in 2012, and all those with stage III nodal MUP were retrieved and matched with two MKP patients according to the same criteria adopted in the Australian and UK cohorts. Clinical data were collected from medical charts.

### 2.4 Statistical analysis

For each study centre, we described patient characteristics in relation to MUP or MKP status using *χ* ^2^ tests of homogeneity for categorical variables and ANOVA for continuous. Likely cutaneous sites of origin of stage III MUPs were imputed from their two matched MKPs separately for study countries, giving two possible sites for each MUP from each centre.

## 3 RESULTS

### 3.1 Australia

There were 82 MUP and 399 MKP patients in the study database^11^ suitable for matching. A total of 156 MKP patients were matched to 80 MUP patients (Table 1): 2 MKPs each for 76 MUP patients, 1 MKP each for 4 MUPs (all female), with no match identified for 2 MUP patients (female, 21 years old, inguinal LND; female, 83 years, cervical LND). LND sites of the 80 MUP patients (mean age 57, 71% male) were evenly distributed across cervical, axillary and inguinal basins (Table 1). When aligned with the LND sites of matched MKP patients, the most common primary cutaneous sites of matched MKPs overall were trunk and legs, with arms the least common (Table S1).

**TABLE 1:** Characteristics of melanoma patients in Australia, the United Kingdom and Italy with a known (MKP) or unknown (MUP) primary site: MKP patients matched to MUP patients by 5‐year age, sex and site of lymph node metastasis

	Australia			UK			Italy		
	MKP (n = 156)	MUP (n = 80)	*P*‐value	MKP (n = 34)	MUP (n = 17)	*P*‐value	MKP (n = 22)	MUP (n = 11)	*P*‐value
	N (%)	N (%)		N (%)	N (%)		N (%)	N (%)	
Sex									
Male	114 (73)	57 (71)	.77	30 (88)	15 (88)	1.00	10 (45)	5 (45)	
Female	42 (27)	23 (29)		4 (12)	2 (12)		12 (55)	6 (55)	1.00
Age, years^a^									
<40 years	27 (17)	15 (19)	.77	9 (26)	3 (18)	.76	3 (14)	2 (18)	.94
40‐59	54 (35)	24 (30)		8 (24)	4 (24)		11 (50)	5 (45)	
60+	75 (48)	41 (51)		17 (50)	10 (59)		8 (36)	4 (36)	
Age, years^a^ (mean ± *SD*)	57 (±16)	57 (±16)	.95	55 (±16)	56 (±16)	.78	59 (±18)	60 (±18)	.84
Site of primary melanoma									
Head and neck	31 (20)	—		4 (12)	—		0 (0)	—	
Arm	11 (7)	—		16 (47)	—		7 (32)	—	
Leg	51 (33)	—		6 (18)	—		5 (23)	—	
Trunk	63 (40)	—		8 (24)	—		10 (45)	—	
Breslow thickness									
T1: 0.2‐1.0 mm	31 (20)	—		9 (26)	—		0 (0)	—	
T2: >1.0‐2.0	61 (39)	—		8 (24)	—		7 (32)	—	
T3: >2.0‐4.0	29 (19)	—		10 (29)	—		8 (36)	—	
T4: >4.0	29 (19)	—		4 (12)	—		7 (32)	—	
Missing	6 (4)			3 (9)					
Site of lymph node metastasis^b^									
Cervical	50 (32)	26 (33)	.99	4 (12)	2 (12)	1.00	0 (0)	0 (0)	1.00
Axilla^c^	51 (33)	26 (33)		24 (71)	12 (71)		14 (64)	7 (64)	
Inguinal	55 (35)	28 (35)		6 (18)	3 (18)		8 (36)	4 (36)	

^a^ Age at lymph node dissection.

^b^ Site of lymph node dissection.

^c^ One MKP with axillary LND had primary site recorded as the neck, assumed to be at the base of the posterior neck.

**FIGURE 1:**
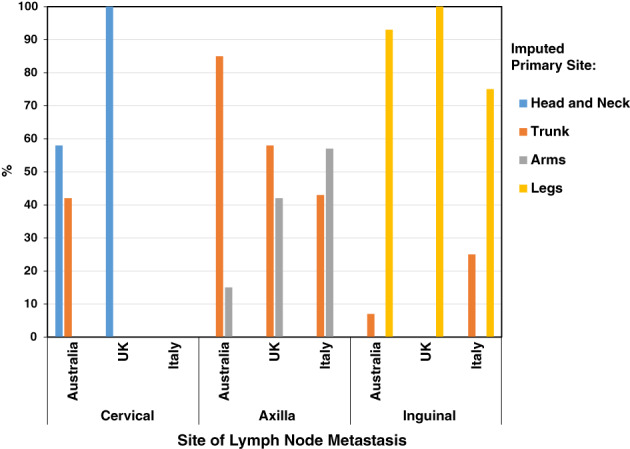
Distribution of imputed primary sites according to MUP site of lymph node metastasis and by country [Color figure can be viewed at wileyonlinelibrary.com]

The most frequently imputed sites for MUPs with axillary LNs were the trunk on the first match (Figure 1) and for MUPs overall (44%) (Table S2), and similarly on the second match (37% of all MUPs) (Table S3). As expected, the legs were the main imputed site of origin for MUPs presenting with inguinal lymphadenopathy, and head and neck for MUPs with cervical lymphadenopathy (Figure 1). Only a minority of MUPs with palpable axillary LNs were estimated to arise on the arms (Figure 1; Tables S2 and S3).

### 3.2 The United Kingdom

In the Manchester database, a total of 18 stage III MUP patients were identified and matched with 34 MKP patients (Table 1). No control was identified for a 33‐year‐old patient who underwent neck LND. The mean age of MUP patients was 56 years and the majority (88%) were male. The majority (71%) presented with axillary adenopathy, with the remainder evenly distributed between cervical and inguinal basins (Table 1). This resulted in a predominance of primary cutaneous sites of matched MKP patients on the arms, followed by the trunk and legs, with fewest on head and neck (Table S1).

The most frequently imputed site for MUPs overall, and for MUPs with axillary lymphadenopathy was again the trunk on the first match (41% and 58%, respectively) followed by the arms (29% and 42%, respectively) (Figure 1; Table S2), with arms the main imputed (61%) site on the second match (Table S3), followed by legs (18%, both matches) and head and neck. Only 6% of MUPs were imputed to arise on the trunk in the second match (Table S3).

### 3.3 Italy

In the Padova database, 11 patients with nodal MUP (mean age 60 years, 45% male) were identified and matched to 22 MKP patients (Table 1). Like the UK series, two‐thirds of the MUP patients presented with axillary lymphadenopathy, and the remaining third with inguinal metastases (Table 1). As a result, most primary cutaneous sites of matched MKP patients arose on the trunk, followed by arms and legs (23%) and none on the head and neck (Table S1). Major imputed sites for MUPs were again the trunk (36% and 55% on first and second matches, respectively) and the arms (36% and 27%), followed by legs (27% and 18%) (Tables S2 and S3).

## 4 DISCUSSION

Based on molecular evidence indicating that MUPs arise from skin,^10^ we aimed to test the study hypothesis that sites of MUPs are disproportionately on the trunk and lower limbs by inferring the anatomic site of origin of MUPs from the corresponding primary sites of matched MKP patients.

Results were consistent with the trunk as the origin of roughly a third to a half of MUPs across the three cohorts (except for the second match in the United Kingdom that yielded a low estimated proportion of MUPs on the trunk—considered an outlier in the context of all other estimates).

However, the next most common inferred sites of MUPs varied by country. In the Australian cohort, the legs accounted for a third of MUPs and the head and neck, for around 20%, with less than 10% estimated to arise on the arms. In contrast, MUPs in the United Kingdom and Italian cohorts were imputed to arise on the arms and then the legs. In the UK cohort, we inferred that the head and neck region accounted for 12% of MUPs, similar to Australia, while no stage III MUPs in Italy occurred on the head and neck since none presented with cervical lymphadenopathy in the study period. Thus the greatest difference between study centres was the low proportion of MUPs inferred to arise on the arms in the Australian compared to the European series. These differences may be climate‐ and clothing‐related: the arms are more likely to be exposed almost year‐round in subtropical Queensland (latitude 27°S) and thus are more visible to both patients and clinicians, while in more temperate Padova and Manchester (latitudes 45°N and 53°N, respectively), arms are covered during most of the year, thus increasing the chance of missing suspicious lesions.

Much of the skin on the head and neck is easy to inspect, yet the head and neck accounted for a small minority (less than a fifth) of inferred sites of origin in the Australian and UK cohorts (the lack in the Italian is likely due to the small sample size).This apparent paradox may be partly explained by the predilection of melanoma and therefore MUPs for subsites less visible, like the ears, scalp and neck, especially in older men who are most affected by MUP.^13^ Indeed, several cases in the Italian series initially had been diagnosed as MUPs but were subsequently found to have either scalp or ear primaries and so were excluded from the study.

A systematic review^8^ has shown that most MUP diagnoses occur in the 50 to 69 year age group and that males are twice as likely as females to be diagnosed with MUP, features confirmed in the present study, apart from the Italian cohort (again, likely due to small numbers, although other explanations such as different cultural norms are also possible). These findings are consistent with the epidemiological features of thick (advanced) primary cutaneous melanoma,^14^, ^15^ the male predominance attributed to the greater delay among men in seeking attention for suspicious skin lesions.^1^

Moreover, as melanoma detection has improved steadily over the last few decades, as evidenced by increasing incidence of thin primaries,^16^ the relative incidence of MUPs has also decreased, from 5.1% in studies before 1980, to 2.7% in those conducted after 1980.^8^ In further support of late detection of primary tumours playing a major role in MUPs, is the strong association between unknown primary cancers in general and low health literacy,^17^ suggesting that health education is a necessary step towards earlier diagnosis and treatment before metastases occur.

Our study was limited by its reliance on clinical databases and medical records to identify cases of both MUPs and MKPs, introducing potential errors in the matching due to inaccuracy and incompleteness of available information. Furthermore, the sample sizes of the European series were small, thus some of the inferred MUP sites of origin were imprecise, being affected by chance distributions of primary melanoma sites in the matched MKP patients. It would have been of interest to compare oncogenic driver mutations between MUPs and their MKP controls, but this was not possible due to a clear bias towards assessing mutation status in MUPs but not MKPs.

We acknowledge that patterns of lymphatic drainage from the skin can vary, adding to imprecision of inferred sites of primary melanomas. On the other hand, our study is entirely novel in its aim to build on the recent molecular evidence^10^ that clinches the empirical evidence^1^ that most MUPs arise on the skin, by imputing the likely skin sites of origin of nodal MUPs, the commonest stage of MUPs at diagnosis. In addition, we used data from three countries, allowing us to generalise results that the trunk is a likely site of origin of MUPs across diverse populations, partly supporting the study hypothesis.

Unexpectedly, the arms were also found to be a likely source of MUPs in European (but not Australian) patients, and the head and neck, despite being largely accessible to inspection, resulted in a site of origin of a proportion of MUPs.

Future studies with larger numbers of patients and participating centres from different latitudes are needed to confirm our results and specify with more precision the skin sites deserving increased scrutiny to enable early detection of primary melanomas.

In conclusion, by assuming that nodal MUPs arise on the skin,^10^ we found that the trunk is the most likely site of origin of these tumours across various populations. Additionally, the arms may give rise to more than 40% of MUPs with axillary node involvement in European populations living in temperate climates.

These results underscore the need for regular and thorough skin examinations, especially in males, and for increased health literacy, to improve early detection of cutaneous melanoma and reduce the risk of metastatic disease at the time of presentation.

## CONFLICT OF INTEREST

The authors declare no conflicts of interest.

## AUTHOR CONTRIBUTIONS

The work reported in the article has been performed by the authors unless clearly specified in the text. Study conception and design: Adele C. Green, Deemesh Oudit. Acquisition of the data: Bethan Clayton, Ferhan Muneeb, Megan E. Grant, Kiarash Khosrotehrani, B. Mark Smithers, Romina Spina, Luca G. Campana. Analysis of the data: Maria Celia B. Hughes, Adele C. Green. Interpretation of the data: All authors. Writing the article: Bethan Clayton, Adele Green. Critical revision of the article: All authors. Approval of final article and the decision to submit the article: All authors.

## ETHICS STATEMENT

Ethics approval was obtained for each cohort respectively: UK (reference: 16/LO/0387); Australia (reference: P3610); Italy (reference CESC IOV 2020/36). Informed consent was not required as studies were based on clinical registers of anonymised patient data.

## Supporting information


**Appendix S1**Supporting Information.

## Data Availability

The data that support the findings of our study are available from the corresponding author upon reasonable request.
