# Supplementary material for: Hypothesised cutaneous sites of origin of stage III melanomas with unknown primary: A multicentre study
Source: Int J Cancer. 2022 Apr 25;151(3):396–401. doi: 10.1002/ijc.34020 (PMC9325056; doi:10.1002/ijc.34020)
Supplement: Supplementary file 1 — Appendix S1Supporting Information. [file IJC-151-396-s001.pdf]

Hypothesised cutaneous sites of origin of stage III melanomas with unknown primary: a multi-centre study

Bethan Clayton, Ferhan Muneeb, Maria Celia B Hughes, Megan E. Grant, Kiarash Khosrotehrani, B Mark Smithers, Romina Spina, Luca G Campana, Deemesh Oudit, Adele C. Green

Table of Contents

Supplementary Table 1. Primary site and site of lymph node metastasis for patients with known primary (MKPs) 2

Supplementary Table 2. Site of primary melanoma imputed for MUPs based on primary sites of MKPs matched by 5-year age, sex and LND site, first match 3

Supplementary Table 3. Site of primary melanoma imputed for MUPs based on primary sites of MKPs matched by 5-year age, sex and LND site, second match 4

Supplementary Table 1. Primary site and site of lymph node metastasis for patients with known primary (MKPs)

MKP Primary Site	MKP Site of lymph node metastasis			
	Cervical	Axilla	Inguinal	Total
Australia				
Head & Neck	31 (62)	0 (0)	0 (0)	31 (21)
Arms	0 (0)	11 (22)	0 (0)	11 (7)
Legs	0 (0)	0 (0)	51 (93)	51 (33)
Trunk	19 (38)	40 (78)	4 (7)	63 (40)
**Total**	50 (32)	51 (33)	55 (35)	156 (100)
UK				
Head & Neck	4 (100)	0 (0)	0 (0)	4 (12)
Arms	0 (0)	16 (67)	0 (0)	16 (47)
Legs	0 (0)	0 (0)	6 (100)	6 (18)
Trunk	0 (0)	8 (33)	0 (0)	8 (24)
**Total**	4 (12)	24 (71)	6 (18)	34 (100)
Italy				
Head & Neck	0 (0)	0 (0)	0 (0)	0 (0)
Arms	0 (0)	7 (50)	0 (0)	7 (32)
Legs	0 (0)	0 (0)	5 (63)	5 (23)
Trunk	0 (0)	7 (50)	3 (38)	10 (45)
**Total**	0 (0)	14 (64)	8 (36)	22 (100)

Supplementary Table 2. Site of primary melanoma imputed for MUPs based on primary sites of MKPs matched by 5-year age, sex and LND site, first match

Imputed Primary Site	MUP Dissection Site			Total
	Cervical	Axilla	Inguinal	
Australia				
Head & Neck	15 (58)	0 (0)	0 (0)	15 (19)
Arms	0 (0)	4 (15)	0 (0)	4 (5)
Legs	0 (0)	0 (0)	26 (93)	26 (33)
Trunk	11 (42)	22 (85)	2 (7)	35 (44)
**Total**	26 (33)	26 (33)	28 (35)	80 (100)
UK				
Head & Neck	2 (100)	0 (0)	0 (0)	2 (12)
Arms	0 (0)	5 (42)	0 (0)	5 (29)
Legs	0 (0)	0 (0)	3 (100)	3 (18)
Trunk	0 (0)	7 (58)	0 (0)	7 (41)
**Total**	2 (12)	12 (71)	3 (18)	17 (100)
Italy				
Head & Neck	0 (0)	0 (0)	0 (0)	0 (0)
Arms	0 (0)	4 (57)	0 (0)	4 (36)
Legs	0 (0)	0 (0)	3 (75)	3 (27)
Trunk	0 (0)	3 (27)	1 (25)	4 (36)
**Total**	0 (0)	7 (64)	4 (36)	11 (100)

Supplementary Table 3. Site of primary melanoma imputed for MUPs based on primary sites of MKPs matched by 5-year age, sex and LND site, second match

Imputed Primary Site	MUP Site of lymph node metastasis			
	**Cervical**	**Axilla**	**Inguinal**	**Total**
Australia				
Head & Neck	16 (67)	0 (0)	0 (0)	16 (21)
Arms	0 (0)	7 (28)	0 (0)	7 (9)
Legs	0 (0)	0 (0)	25 (93)	25 (33)
Trunk	8 (33)	18 (72)	2 (7)	28 (37)
**Total**	24 (32)	25 (33)	27 (36)	76[1] (100)
UK				
Head & Neck	2 (100)	0 (0)	0 (0)	2 (12)
Arms	0 (0)	11 (92)	0 (0)	11 (65)
Legs	0 (0)	0 (0)	3 (100)	3 (18)
Trunk	0 (0)	1 (8)	0 (0)	1 (6)
**Total**	2 (12)	12 (71)	3 (18)	17 (100)
Italy				
Head & Neck	0 (0)	0 (0)	0 (0)	0 (0)
Arms	0 (0)	3 (43)	0 (0)	3 (27)
Legs	0 (0)	0 (0)	2 (50)	2 (18)
Trunk	0 (0)	4 (57)	2 (50)	6 (55)
**Total**	0 (0)	7 (64)	4 (36)	11 (100)

[1]Total is 76 since n=4 MUPs matched to only 1 MKP
